# Consented indicators for the evaluation of integrated strategies of community health promotion targeting children and adolescents: results of an eDelphi

**DOI:** 10.1186/s12889-023-17370-4

**Published:** 2024-01-22

**Authors:** Myriam Robert, Michaela Coenen, Julia Bauer, Stephan Voss, Caroline Jung-Sievers

**Affiliations:** 1 Chair of Public Health and Health Services Research, Institute for Medical Information Processing, Biometry, and Epidemiology (IBE), Faculty of Medicine, Ludwig Maximilian University (LMU) of Munich, Munich, Germany; 2 Pettenkofer School of Public Health, Munich, Germany

**Keywords:** Health promotion, Prevention, Evaluation, Delphi method, Health status indicator, Child, Infant, Integrated community-based interventions

## Abstract

**Background:**

To date, there is no consensus on indicators for the evaluation of integrated community-based interventions for health promotion and prevention targeting children and adolescents. This study aims at consenting on a scoped set of indicators to evaluate integrated community-based interventions.

**Methods:**

Out of 738 indicators derived from a literature search, we preselected 94 indicators allotted to 20 domains based on an internal quality appraisal and consensus process and conducted an eDelphi procedure to assess their relevance in view of experts. Experts were recruited in the field of public health, health sciences and communal health promotion in practice and were invited as participants in this eDelphi. During the eDelphi, 47 experts rated the relevance of 94 indicators in two rounds. Consensus was defined as agreement of 75% (or above).

**Results:**

After round 1, 27 indicators among 11 consented subdomains reached a consensus on relevance. After round 2, a total of 36 indicators reached consensus on relevance in 9 subdomains (such as socioeconomic factors, health education, nutrition and physical activity, oral health, overall health status, specific health conditions, drug related behavior, exposure to drugs and violence, family factors).

**Conclusions:**

These identified indicators may provide a basis for evaluation concepts of integrated community-based interventions for children and adolescents to inform stakeholders about intervention impacts.

**Supplementary Information:**

The online version contains supplementary material available at 10.1186/s12889-023-17370-4.

## Background

Promoting health for children and adolescents is an essential task in public health, as it may not only impact the current population but also represents an important investment into future generations [1]. In times of social, environmental and economic crises, families with children are often particularly vulnerable [2]. Therefore, health promotion and prevention strategies targeting children and adolescents and their families are of utmost importance.

Setting approaches have been proven to be effective modes of delivery for health promotion and prevention strategies. Especially, the community and/or municipal setting has received considerable attention over the last decades resulting in various initiatives, structures and funding sources [3]. On the one hand, community-based interventions have several advantages: they may integrate multiple sectors beyond the health sector, make use of co-benefits and influence contextual factors as well as target the general population and involve different risk groups [4]. On the other hand, interventions are often complex and their impact is difficult to assign to and to evaluate.

Complex interventions [5] are implemented in various settings and are labeled differently depending on the specific field of application. Complex interventions in the community setting that include actors from different sectors can be referred to as *Integrated Community-Based Interventions or approaches* [6]. Focusing on health promotion and prevention, these interventions are also referred to as *Integrated Strategies of Community Health Promotion* (ISCHP) in different contexts [7]. ISCHP are characterized by an overall health promotion approach through the collaboration of communal actors from usually separate institutions and sectors [8]. Based on this collaboration, ISCHP aim to enhance the living conditions of members of the community and to promote health with an additional focus on socially disadvantaged groups [8]. ISCHP might target all age groups, but ISCHP for children and adolescents are often applied and common [9].

A specific national German example for ISCHP are the so called “*Präventionsketten”* (translated as “preventions chains”). *“Präventionsketten”* are networks that regulate interdisciplinary cooperation in a binding manner. Services from, for instance, the fields of education, health and social services in a community setting coordinate themselves to co-develop according to expressed residents’ needs. The focus is on phases of biographical transitions in children and adolescents, for example from nursery to primary school or from primary to secondary school [10].

Up to date, the evidence base of impact evaluations of community strategies for health promotion and prevention for children and adolescents (or *ISCHP* or more specifically “*Präventionsketten”*) is scarce [9]. Although indicators can be used to monitor child and adolescent health to evaluate the intervention’s results, up to date there is no consensus on how to select health indicators [11], and which indicators should be used for the evaluation of ISCHP (such as “Präventionsketten”) [1, 11, 12].

In this study, we aimed to identify a set of expert-based, pragmatic and real-world indicators to evaluate ISCHP targeting children and adolescents based on expert feedback from an interdisciplinary background in practice and research using an eDelphi method. The results of this approach may inform stakeholders for planning and performing evaluations in the field.

## Methods

### Study design

We used a multi-step procedure including (i) a scoping review of the literature to identify potential relevant indicators (see Selmani et al., [13]) ii) a structured preselection of indicators based on internal quality appraisal, and (iii) an eDelphi study for expert consensus on the final indicators selection.

### Step 1: identification of relevant health indicators by evidence synthesis

**Fig. 1:**
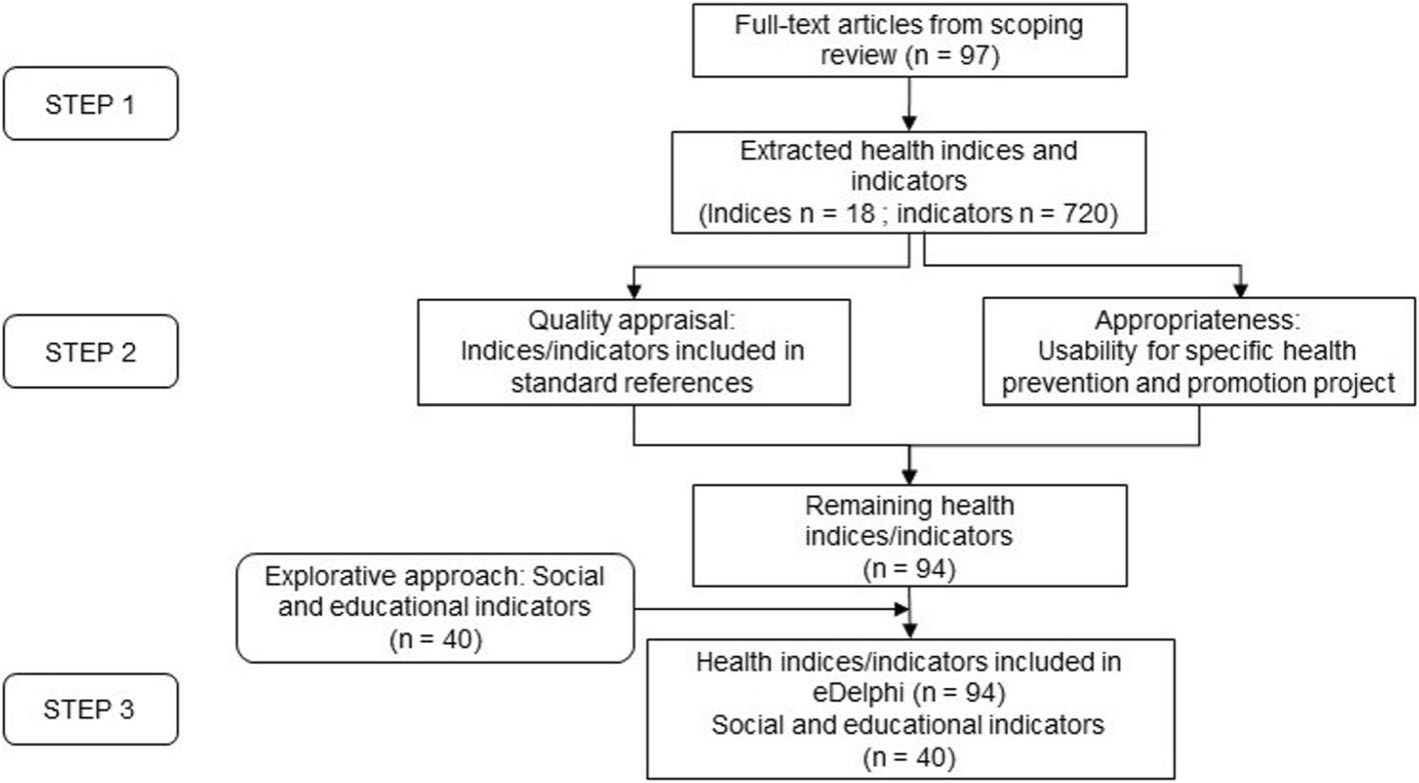
Flow chart of identification and preselection of indicators for the eDelphi

The identification and selection process is shown in Fig. 1. Health indicators were identified by a literature search for a scoping protocol published by our group in 2021 reporting on health indices [13]. All articles identified in the scoping review including indices were additionally screened for potential single health indicators. Adapted inclusion and exclusion criteria were used (for details see Additional file Table A.1).

### Step 2: preselection of health indicators

To condense the identified indicators to a number of approximately 100 indicators, we performed internal quality appraisal by applying established indicators lists and frameworks [14, 15] through internal team review. First, we compared the indicators to five references for health indicators [14, 16–19]. Second, two authors (CJS, SV) independently assessed each indicator’s usability for the evaluation of ISCHP targeting children and adolescents (and in doubt, more specifically, *Präventionsketten)* [20]. A third author resolved discrepancies (MC). Indicators were then clustered in 20 thematic distinct subdomains. Social and educational indicators that could be used for evaluating ISCHP targeting children and adolescents were not included into the structured eDelphi to assure focus and feasibility, but were assessed in an exploratory manner (providing a list of indicators from OECD reports proposed to participants as an orientation to comment on social and educational aspects and indicators) [21, 22].

### Step 3: expert consensus via the eDelphi method

#### Online delphi procedure

We used the Delphi method to assess the relevance of indicator subdomains and single indicators for child and adolescent health. The Delphi method is appropriate for complex questions characterized by uncertainty and missing evidence [23] and is an established procedure to select health and other indicators for different purposes [15, 24]. It aims at building a consensus by collecting expert opinions when a higher evidence level is not possible to achieve [25]. A Delphi procedure typically (i) is anonymous, (ii) takes place in several rounds, and (iii) informs participants about results of previous round(s) and gives them the opportunity to change their responses [23]. We chose the online format (eDelphi) to facilitate implementation (in time of the COVID pandemic) [25]. The software used was LimeSurvey [26].

#### Survey development

**Fig. 2:**
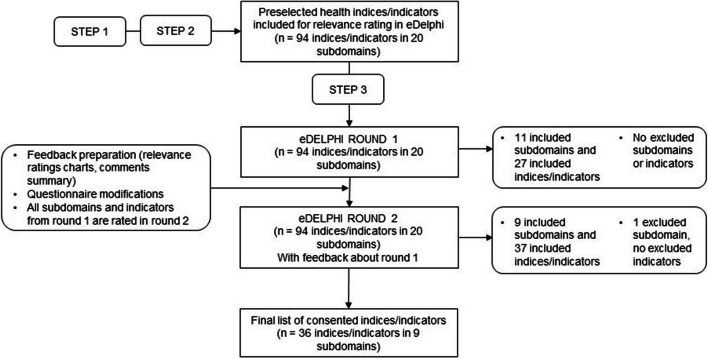
Steps taken from the initial eDelphi questionnaire to the final indicators list

This eDelphi process consisted of two rounds in which experts rated the relevance in terms of an essential aspect to be considered in the evaluation of ISCHP (indicator subdomains and single indicators) in an online survey. The survey was piloted within the research team and feedback was incorporated in the final version. Figure 2 highlights the steps taken from the initial eDelphi questionnaire to the final indicators list. There were small adaptations in the survey from round 1 to round 2 based on panelists’ feedback and piloting (deletion of one response option concerning the professional background of panelists for data protection reasons; changes in wording and order of indicators).

#### Expert panel selection and recruitment

Eligible experts contacted for the eDelphi were (i) experts with public health research expertise in the fields of “health promotion and prevention”, “child health” and/or “health reporting”, and (ii) practitioners of municipal strategies for health promotion and prevention for children and adolescents or integrated community-based interventions to promote health in children and adolescents (mainly, *Präventionsketten* in Germany). Experts were identified via internet search and contact data was found in publicly available sources or via the snowballing technique, i.e. by asking the experts to propose other possible participants (primarily directed to, but not restricted to the German expert community and the national context). Invitation emails were sent to all potential participants. We aimed at a sample size of about 20 participants [23].

#### Analyses within the eDelphi

##### Round 1

First, panelists rated the relevance of each subdomain (“*yes”/”no*”). Only if a subdomain was considered as relevant, the associated indicators were presented. Each indicator’s relevance was rated on a 5-point Likert-scale from 1 = “*not relevant*” to 5 = “*very relevant*”. The option 0 = “*not specified*” was also given for each indicator. A description of each indicator was provided in the survey. Panelists could leave comments after each subdomain and its associated indicators to explain their decisions and to suggest additional indicators.

After completion of round 1, the mean relevance of each subdomain and of each indicator was calculated in order to assess if a consensus was reached concerning an indicator’s (ir)relevance. Consensus was reached if ≥ 75% of the experts came to the same decision. As defined a priori [27], *consensus on relevance* for an indicator was reached if (i) ≥ 75% of the experts considered the associated subdomain as relevant (“*yes*”), and (ii) ≥ 75% of the experts rated an indicator as 4 = “*relevant*” or 5 = “*very relevant*”. Respectively, *consensus on irrelevance* for an indicator was reached if (i) ≥ 75% of the experts considered the associated subdomain as irrelevant (“*no*”), and (ii) ≥ 75% of the experts rated an indicator as 1 = “*not relevant*” or 2 = “*little relevant*”. Comments were summarized and used for adaptation of the survey for round 2. Participants in round 2 received the feedback from round 1 in the form of graphs with the distribution of the panelist’s answers showing the results of the 5-point Likert scale. Additionally, free text comments were provided. For further details regarding included and excluded information, see also Fig. 2.

##### Round 2

It was defined a priori that no indicator was to be included in round 2 that was rated to be irrelevant in round 1 [27]. At the beginning of round 2 and with each item, panelists were shown the anonymized and pooled results of round 1 via graphical and written summaries. They then again rated the relevance of the subdomains and indicators. The final list consisted of subdomains and indicators which both achieved consensus on relevance. Frequency calculations of each subdomain and of each indicator were carried out using SPSS 27.0. Free-text comments were analyzed using MAXQDA [28].

### Ethics statement

The study was approved by the Ethics Committee of the Faculty of Medicine at LMU Munich (project number: 21–0767). Before participation, all experts were informed comprehensively about the study goals and procedure and gave their informed consent. The data of this study was stored and processed anonymously. The participants’ identity was not known to the other participants. It was possible to tick a box if personalized acknowledgement would be appreciated.

## Results

### Step 1: identification of relevant health indicators by evidence synthesis

In total 720 indicators and 18 indices were extracted in our first step based on our search strategy as described previously [13]. The extracted indicators were categorized into the following indicator domains: “health status”, “health determinants”, “service coverage” and “health system and policy” according to previous classifications [1, 14]. Most of the indicators were related to “health determinants” (52%) and “health status” (38%). “Service coverage” (4%) and “health system and policy” (5%) were less represented.

### Step 2: preselection of indicators

Within step 2, a set of 94 indicators was consented for the eDelphi, including all 4 domains mentioned above. The list developed from OECD reports to guide the participants by suggesting social and educational indicators comprised 40 social and educational indicators (see Additional file Table A.2).

### Step 3: expert consensus via eDelphi method

#### Participants

Out of 283 experts who were invited to the eDelphi (32 of them identified via snowballing), 62 agreed to participate. The first round was completed by 55 of those 62 experts (89%). Most of the experts were female (82%) and worked in the broader context of *Präventionsketten* (44%), followed by experts in public health research (27%) and experts working in politics, administration or health departments (22%; see Table 1).

**Table 1:** Characteristics of the expert panel

Characteristics	Round 1		Round 2	
**Participants** n	55		47	
**Sex** n (%)				
Female	45	(82%)	37	(79%)
Male	10	(18%)	10	(21%)
**Age in years** n (%)				
18–29	1	(2%)	1	(2%)
30–49	31	(56%)	24	(51%)
50–69	21	(38%)	20	(43%)
≥ 70	2	(4%)	2	(4%)
**Profession** ^**1**^ n (%)				
Public health research: prevention and health promotion, child health or health reporting	15	(27%)	11	(23%)
Politics/administration/health department	12	(22%)	17	(36%)
Participation in “Präventionsketten”^2^	24	(44%)	19	(40%)
Participation in other Integrated Strategies of Community Health Promotion (not “Präventionsketten”)	2	(4%)	0	
Other	2	(4%)	0	

*Notes.* ^1^ In round 1, we distinguished between participation in the specific prevention chain in Munich and participation in other prevention chains in Germany. To ensure the anonymity of the few participants, we have dispensed with this distinction in round 2. ^2^ A specific national German example for *Integrated Strategies of Community Health Promotion* (ISCHP) are the so called “*Präventionsketten”* (translated as “preventions chains”)

#### Indicator ratings

In round 1, 11 of the 20 subdomains of indicators reached a consensus on relevance (rated as *relevant* by ≥ 75% of the rating panelists): socioeconomic factors, health education, nutrition and physical activity, health status (physical/mental health conditions), drug related behavior, overall health status, oral health, health behavior (multiple factors), exposure to drugs and violence, family factors, functional health status (sorted in descending order of consensus on relevance after round 1; see Additional file Table A.3). Among these consented subdomains, 27 indicators reached a consensus on relevance (considered *relevant* or *very relevant* by ≥ 75% of the panelists that rated the subdomain as *relevant*). No subdomain and no indicator reached a consensus on irrelevance (rated as *little relevant* or *not relevant* by ≥ 75% of the panelists). According to the methodology agreed before conducting the study, all indicators of round 1 were included again, all 20 subdomains and 94 indicators of round 1 were included in round 2. Table A.4 (Additional file) provides an overview of the ratings of subdomains (only) in rounds 1 and 2.

The second round was completed by 47 of 55 experts (86%) who participated in round 1. In this round, only 9 subdomains reached a consensus on relevance: socioeconomic factors, nutrition and physical activity, health education, oral health, overall health status, health status (physical/mental health conditions), drug related behavior, exposure to drugs and violence, family factors (sorted in descending order of consensus on relevance after round 2; Additional file Table A.4 for subdomains’ ratings after round 1 and 2; Additional file Table A.5 for subdomains and indicators’ ratings after round 2). Among the subdomains agreed on their relevance, a final list of 36 associated indicators reached a consensus on relevance (Table 2). One subdomain (vital and laboratory parameters) was rated to be *not relevant* by 83% of the panelists and therefore reached a consensus on irrelevance; indeed, it seemed too specific and not matching with potential ISCHP effects, according to the panelists’ comments. Apart from that, no single indicator reached a consensus on irrelevance.

**Table 2:** List of indicator subdomains and associated indicators which reached consensus on relevance after round 2

Domain	Subdomain (consensus level on relevance^1^)	Indicators	Consensus level on relevance^1^	N	Median
Health determinants	**Socioeconomic factors (100%)**				
		Proportion of early school leavers	89%	42/47	5
		Percentage of women and children with inadequate social support	89%	42/47	5
		Perceived social support at the individual level	89%	42/47	5
		Child poverty rate	87%	41/47	5
		Children with supportive neighborhood	85%	40/47	5
Health determinants	**Nutrition and physical activity (100%)**				
		Physical activity	96%	45/47	4
		Physical inactivity	94%	44/47	5
		Physical activity as organized physical activity	92%	43/47	4
		Sedentary behavior	89%	42/47	5
		Nutritional behavior	89%	42/47	4
Health systems/policy	**Health education (100%)**				
		Preventive oral health programs in kindergartens^2^	87%	41/47	4
Health status	**Oral health (98%)**				
		DMFT (decayed, missing, filled, tooth) index	87%	41/47	4
Health status	**Overall health status (94%)**				
		Perceived overall health status	80%	36/45	4
Health status	**Health status as specific physical, mental health conditions (88%)**				
		Children with developmental delay	100%	42/42	5
		Refusal to attend school	95%	40/42	5
		Depression	88%	37/42	5
		Emotional distress	88%	37/42	4
		Subjective health complaints	83%	35/42	4
		Eating disorder	83%	35/42	4
Health determinants	**Drug related behavior (88%)**				
		Current alcohol consumption	100%	42/42	5
		Extreme/harmful alcohol consumption	98%	41/42	5
		Illicit drug dependence	95%	40/42	4
		Current overall tobacco use	93%	39/42	4
		Alcohol dependence	88%	37/42	4
		First cigarette smoking before age 13 years	88%	37/42	4
		Total alcohol consumption	88%	37/42	5
		First alcohol consumption before age 13 years	86%	36/42	4
		Tobacco dependence	81%	34/42	4
Health determinants	**Exposure to drugs and violence (83%)**				
		Children in smoking household	85%	34/40	5
		Number of children reported abused or neglected	85%	34/40	5
		Substantiated child maltreatment including experience of physical abuse, neglect or deprivation of necessities, medical neglect, sexual abuse, psychological or emotional maltreatment	85%	34/40	5
		Exposure to physical violence in the community	83%	33/40	5
		Intimate partner violence, injury, physical or sexual abuse	80%	32/40	5
		Children who had ever been physically forced to have sexual intercourse when they did not want	78%	31/40	5
Health determinants	**Family factors (79%)**				
		Smoking during pregnancy	87%	32/37	5
		Adult overweight or obesity	78%	29/37	4

*Notes*. ^1^ Percentage of panelists who rated the subdomain or indicator as *relevant* or *very relevant*, incl. *not specified* responses. ^2^ This indicator was identified through literature research. Although this indicator does not fit into the row of identified outcome indicators as reported within the other subdomains, we decided to report it nevertheless according to our eDelphi protocol

#### Open feedback

Many participants used the free text fields to express their opinion on subdomains and indicators, e.g., regarding the indicators’ quality, the reasons of relevance or irrelevance, improvements in formulation, data availability challenges or possible additional indicators. Some examples of these additional suggestions are provided in Table A.6 (Additional file). They also mentioned possible data sources, e.g., school entry examination which is mandatory in all federal states in Germany.

Overall, while in this eDelphi only health indicators could be rated, the experts’ comments pointed out specifically to the importance of social and educational indicators to evaluate municipal strategies or integrated community-based interventions to promote health in children and adolescents: “*I consider it obligatory to stronger include (…) the social area, dealing with ‘Präventionsketten*’; “*Precisely this interconnectedness [between health indicators] and the social and educational fields is a central element of communal ‘Präventionsketten*’” (all quotes were translated by the authors). However, there was disagreement between the panelists whether socioeconomic factors (e.g., child poverty rate) could be changed by these interventions and could therefore be an indicator to evaluate. Some panelists argued that prevention should not aim at reducing the poverty rate, but rather target its consequences.

Furthermore, some experts’ comments indicated concern about the suitability of individual health status indicators to evaluate ISCHP, as ISCHP mainly intervene on municipal structures and processes: “*Since ‘Präventionsketten’ strongly tend to a structural prevention approach (…), the causality of effects on the individual health status is very difficult to establish or sometimes not possible to deduce. Besides, the results on the children’s individual level are only to expect after a long period of time*”. This was also reflected by comments of participants missing short-term indicators on direct effects of “*Präventionsketten*“: “*First, the focus should be on structural changes that were caused by the work of ‘Präventionsketten’”; “For example, quality of networking, changes in (…) administrative processes, target group participation, changes/improvements in the living conditions/setting of a district*”.

Sometimes, the comments expressed disagreement (e.g., about the advantages and the drawbacks of indices merging several aspects into one single measure).

For some experts, the subdomains’ and indicators’ relevance was difficult to judge, since this depends on each specific intervention in a specific context: “*The relevance highly depends on the objectives of a ‘Präventionskette’*”. Thus, one expert suggested to differentiate between “standard indicators” as best practice indicators and “special indicators” that would be specific for projects with a particular focus or objectives.

## Discussion

Based on results of a previous scoping review and further scoping of single indicators in this project [13], we conducted an eDelphi with a variety of experts and stakeholders in the public health field to select and prioritize child and adolescent health indicators for ISCHPs targeting children and adolescents in Germany (and if applicable, *Präventionsketten* as a specific example). From 94 indicators initially included in the eDelphi, 36 indicators among 9 subdomains were rated as *relevant* or *very relevant* by ≥ 75% of the panelists.

To summarize, the final list of indicators presented here contains aspects that have been applied in previous community health promotion evaluations in Germany (e.g., oral health [29] or drug use [30]). And several additional indicators and data sources suggested by the panelists are in line with the demographic, social, educational, health and contextual aspects provided in other works or recommendations [15, 31, 32]. However, the possibilities to compare our findings with related work of others are limited, since there are to our knowledge no other consensus studies on evaluation indicators for municipal strategies or integrated community-based interventions for promoting health in children and adolescents so far.

The differences of opinions in the comments discussing indices’ appropriateness corroborate with previous assertions depicting an ongoing debate on the use of indices [11].

The ratings and comments underline the importance of assessing socioeconomic factors (100% consensus level on relevance in both rounds). However, there seems to be no consensus on what specific goals are pursued and reachable by municipal strategies or integrated community-based interventions, an ambiguity which is common in health promotion and prevention, but hinders comparability and impact analyses [33]. Promoting a standardization of outcomes as well as a better understanding among stakeholders of the intervention about potential pathways and effects to associated results is needed (i.e., through application of consented and participatively developed logic models). This would also contribute to legitimate indicators necessary for evaluation.

Despite the importance of standardization, we recognize that each intervention will require adaptations to a possible standardized set of indicators. The aim of this study is to propose a set of expert-based, pragmatic indicators that can be used for evaluating municipal strategies or integrated community-based interventions for children, given the fact that no consented set of indicators for this purpose exists. The results of this study may serve as a basis for selecting indicators while considering specific characteristics of the intervention, the context, data availability etc.

Although the literature search found few indicators related to health systems and policy, and consequently only one indicator was included in the eDelphi. However, this indicator could be rather regarded as a process indicator. This item belongs to those with the highest consensus on relevance (100% for the subdomain “health education” after round 2). Therefore, health systems and policy indicators seem to be particularly important while only few indicators exist. This can be regarded as a gap identified by our study. One indicator deemed particularly relevant (preventive oral health programs in kindergartens) is similar to previously consented health promotion indicators [15].

Furthermore, the panelists suggested more direct measures of interventions effects, such as changes in organizational practices that may, for instance, include changes in communication infrastructure or leadership practices [34]. In order to shed light on these changes, we have defined process indicators on the basis of a logical model and collected them in empirical process evaluation study designs (these projects are part of a comprehensive evaluation project of the “Präventionskette Freiham” in Munich, Germany which is currently being evaluated by the Chair of Public Health and Health Services at LMU Munich; data not shown). Developments in the field of evaluations of complex interventions show that often important changes cannot be detected using particular indicators targeting predefined outcomes. Rather particular structural changes maybe better represented by process evaluation and qualitative methods. Aspects of the quality of changes and practices can thus be determined. We therefore propose that a combination of process and outcome evaluation designs is an adequate strategy to get a comprehensive picture of more complex and unanticipated impacts of an intervention [35].

In addition, health promotion and prevention indicators, especially to measure structural changes, are still under development compared to well-established indicators on disease treatment and rehabilitation [12, 15]. The “direct” indicators suggested by the panelists corroborate with some short-term outcomes of the few reported evaluations in Germany (e.g., stronger improvements in intersectoral collaboration for health promotion) [30]. Further tools aiming at monitoring community structures exist and can be considered [32]. Besides, examples of structural indicators are provided by the German national health monitoring institute in a set on child obesity determinants [36], which indeed concords with the panelists’ suggestions (e.g., number of playing areas).

This project mainly investigated health indicators. However, not all participants agreed on merely focusing on health and expressed the need to incorporate other areas of indicators. In addition, the panelists questioned the adequacy of long-term indicators such as health status factors to evaluate municipal strategies or integrated community-based interventions for promoting health for children and adolescents. This is in line with previous recommendations to increasingly focus on proximal, intervention-sensitive indicators rather than distal outcomes such as health status to evaluate community-based health promotion [4]. This also concords with previous, nonscientific municipal strategies or integrated community-based interventions evaluations that put more attention to social and educational outcomes than to health outcomes [31]. Obtained in a non-systematic, explorative approach, the panelists’ comments provide some starting leads on potentially relevant social and educational aspects. As noted by the panelists, further work and complementation with social and educational indicators is necessary. However, long-term indicators like health status measures are the most developed and available data [12, 15], which is also why they are chosen to evaluate municipal strategies or integrated community-based interventions long-term outcomes [7].

### Strengths and limitations

The presented work has several strengths including a structured methodology of the indicators’ identification and selection; a high number of diverse experts with different backgrounds and a high participation rate; as well as the consideration of practical perspectives in a participative approach [25, 27].

The initial search of indicators in a systematic literature review led to a list of indicators focusing on the content rather than on established definitions [15]. This allowed the eDelphi to be accessible to experts with mixed backgrounds, including stakeholders without epidemiological training. This could also improve the feasibility of the survey, which still remained long despite the indicators’ preselection steps [15]. In addition, the collection of panelists’ comments could advert on possibly missing subjects and suggest improvements.

However, there are also several limitations. For instance, as in every consensus building procedure, there is a risk to neglect relevant and unusual opinions [37]. Delphi studies are exposed to experts’ bias, i.e., experts’ opinions are not necessarily the “correct” answers, as well as research bias, i.e., some relevant indicators could have been left out during the eDelphi development [23]. The experts’ recruitment did not aim to be representative. Ultimately, the panel mainly consisted of women involved in *“Präventionsketten“* based in Germany; only few full-time researchers agreed to participate. Notably, only one subdomain and none of the 94 indicators could be excluded by reaching a consensus on irrelevance. Therefore, the results underscore the high number of relevant issues and the complexity of choosing only a limited set of items, an issue that has been described for long [12]. Furthermore, due to a focus on health, there was no structured consensus process for social and educational indicators, and this field was only explored unsystematically. In addition, none of the suggestions for additional indicators has been consented by the experts. This should be considered during further use of the indicator sets and the list of additional suggestions. The diversity of municipal strategies or integrated community-based interventions in children and adolescents made it difficult to have clarity on pursued outcomes. The focus on so-called *“Präventionsketten“* for children and adolescents for recruitment as a specific example appeared necessary but might limit the applicability of the results. Finally, due to the approach chosen for the indicators selection and eDelphi development, the indicators have not yet been defined and operationalized [15]. These are necessary steps of the consequent indicators’ development.

Commonly, further steps follow the indicators’ selection, e.g., the definition and operationalization mentioned above [15]. Another essential aspect that must be investigated is data availability [11], which is still a challenge in German health promotion and prevention [38], in particular on the community level [33]. Therefore, to be able to repeatedly use the indicators, it will be helpful to link indicators to existing data sources, e.g., considering school examinations mentioned by the panelists which were already chosen for previous evaluations (in Germany) [31, 32].

## Conclusions

To our knowledge, this is the first eDelphi study to identify consented child and adolescent health indicators to evaluate municipal strategies or integrated community-based interventions for promoting health in children and adolescents. We consented a set of 36 indicators which reached a consensus on relevance and can therefore be used for designing future evaluations in the field. The identified indicators gather important and diverse aspects of community health promotion for children and adolescents that can be applied to evaluate integrated community-based interventions programs. Furthermore, the experts’ feedback forms a basis for piloting various evaluation projects in the field and points out to remaining challenges. A contribution has been made to inform stakeholders and encourage the further development of an evidence-base on municipal strategies or integrated interventions for children and adolescents in communities.

### Supplementary Information

**Additional file 1: Table A.1 **Inclusion and exclusion criteria for single indicators’ extraction on the basis of the scoping review ^1^ according to the PICo (Population, Interest, Context) scheme. **Table A.2 **List of 40 OECD indicators to guide the participants by suggesting social and educational indicators. **Table A.3** List of indicator subdomains and associated indicators after round 1. **Table A.4** List of indicator subdomains after round 1 and round 2**Table A.5** List of indicator subdomains and associated indicators after round 2. **Table A.6** Examples of additional indicators suggested by panelists in rounds 1 and 2.

## Data Availability

Additional information is available upon request to the corresponding author (CJS).

## Abbreviations

DMFT: Decayed, Missing, Filled Tooth Index
HLI: Healthy Lifestyle Index
ISCHP: Integrated Strategies of Community Health Promotion
OECD: Organization for Economic Co-operation and Development
WHO: World Health Organization

## References

[1] Rigby M (2005). Principles and challenges of child health and safety indicators. Int J Injury Control Saf Promotion.

[2] Aborode AT, Ogunsola SO, Adeyemo AO (2021). A Crisis within a Crisis: COVID-19 and Hunger in African Children. Am J Trop Med Hyg.

[3] Trojan A, Reisig V, Kuhn J (2016). Gesundheitsförderung in Städten Und Gemeinden. Entwicklungsstand Und Perspektiven für das setting „Kommune Nach Verabschiedung Des Präventionsgesetzes [Health promotion in cities and communities. Present state and perspectives for community settings after enacting the Prevention Law in Germany]. Prävention Und Gesundheitsförderung.

[4] Nickel S, von dem Knesebeck O (2020). Effectiveness of Community-Based Health Promotion Interventions in Urban areas: a systematic review. J Community Health.

[5] Craig P, Dieppe P, Macintyre S, Michie S, Nazareth I, Petticrew M (2008). Developing and evaluating complex interventions: the new Medical Research Council guidance. The BMJ.

[6] Mantziki K, Renders CM, Westerman MJ, Mayer J, Borys JM, Seidell JC (2018). Tools for a systematic appraisal of integrated community-based approaches to prevent childhood obesity. BMC Public Health.

[7] Röding D, Walter U, Dreier M (2021). Long-Term effects of Integrated Strategies of Community Health Promotion on Diabetes Mellitus Mortality: a natural policy experiment based on aggregated longitudinal secondary data. J Urb Health.

[8] Böhme C, Reimann B (2018). Integrierte Strategien Kommunaler Gesundheitsförderung. Rahmenbedingungen, Steuerung Und Kooperation.

[9] Böhm K, Gehne D (2018). Vernetzte Kommunale Gesundheitsförderung für Kinder Und Jugendliche. Bundesgesundheitsblatt - Gesundheitsforschung - Gesundheitsschutz.

[10] Richter-Kornweitz A, Utermark K. Werkbuch Präventionskette. Herausforderungen und Chancen beim Aufbau von Präventionsketten in Kommunen. Hannover: Kooperationsverbundes gesundheitliche Chancengleichheit; 2013.

[11] Kohler L (2016). Monitoring children’s health and well-being by indicators and index: apples and oranges or fruit salad? Child: care. Health and Development.

[12] Nutbeam D (1999). Evaluating health promotion. The BMJ.

[13] Selmani A, Coenen M, Voss S, Jung-Sievers C (2021). Health indices for the evaluation and monitoring of health in children and adolescents in prevention and health promotion: a scoping review. BMC Public Health.

[14] World Health Organization. 2018 Global reference list of 100 core health indicators (plus health-related SDGs). World Health Organization; 2018. https://apps.who.int/iris/handle/10665/259951.

[15] Thom J, Mauz E, Peitz D, Kersjes C, Aichberger M, Baumister H (2021). Establishing a Mental Health Surveillance in Germany. Development of a framework concept and indicator set. J Health Monit.

[16] OECD. Health at a Glance 2019: OECD Indicators. Paris: OECD Publishing. ; 2019. Available from: https://www.oecd-ilibrary.org/content/publication/4dd50c09-en [letzter Zugriff 22.09.2021].

[17] Pavarini G, Lorimer J, Manzini A, Goundrey-Smith E, Singh I (2019). Co-producing research with youth: the NeurOx young people’s advisory group model. Health Expectations: An International Journal of Public Participation in Health care and Health Policy.

[18] Arbeitsgemeinschaft der Obersten Landesgesundheitsbehörden. Indikatorensatz für die Gesundheitsberichterstattung der Länder, Vol. 3. Bielefeld: Ministerium für Gesundheit, Soziales, Frauen und Familie des Landes Nordrhein-Westfalen (MGSFF); 2003.

[19] Reisig V, Moritz B, Poppe F, Zollikofer S, Kuhn J (2018). Daten Zur Prävention Und Gesundheitsförderung in Bayern 2018 - Präventionsberichterstattung in Bayern.

[20] Netzwerkmanagement Präventionskette Freiham. Gut und gesund Aufwachsen - Präventionskette Freiham [online Dokument]. inforo. ; 2020 [Available from: https://www.muenchen.de/rathaus/dam/jcr:c6861751-03d8-451a-8166-b7baed07ab3d/Steckbrief_Praeventionskette_Freiham.pdf [letzter Zugriff 22.04.2022].

[21] OECD, Society at a. Glance 2019: OECD Social Indicators. Paris: OECD Publishing; 2019. Available from: https://www.oecd-ilibrary.org/content/publication/soc_glance-2019-en [letzter Zugriff: 22.09.2021].

[22] OECD. Bildung auf einen Blick 2020: OECD-Indikatoren. Bielefeld: wbv Media. ; 2020. Available from: https://www.oecd-ilibrary.org/education/bildung-auf-einen-blick-2020-oecd-indikatoren_6001821nw;jsessionid=IuMPVy18f62Xhu7lTSzxz-Qj.ip-10-240-5-151 [letzter Zugriff 22.09.2021].

[23] Hasson F, Keeney S, McKenna H (2000). Research guidelines for the Delphi survey technique. J Adv Nurs.

[24] Boulkedid R, Abdoul H, Loustau M, Sibony O, Alberti C (2011). Using and reporting the Delphi method for selecting healthcare quality indicators: a systematic review. PLoS ONE.

[25] Häder M (2014). Delphi-Befragungen: Ein Arbeitsbuch.

[26] Limesurvey. Datenschutzhinweise [Webseite]. [Available from: https://www.limesurvey.org/de/datenschutzhinweise [letzter Zugriff 12.07.2021].

[27] Jünger S, Payne SA, Brine J, Radbruch L, Brearley SG (2017). Guidance on conducting and REporting DElphi studies (CREDES) in palliative care: recommendations based on a methodological systematic review. Palliat Med.

[28] Software VERBI (2017). MAXQDA. Software für qualitative Datenanalyse.

[29] Fröhlich-Gildhoff K, Rauh K, Kassel L, Tschuor S, von Hüls B, Döther S (2017). Zwischenbericht Der evaluation des gesamtprojekts Präventionsnetzwerk Ortenaukreis.

[30] Röding D, Soellner R, Reder M, Birgel V, Kleiner C, Stolz M (2021). Study protocol: a non-randomised community trial to evaluate the effectiveness of the communities that care prevention system in Germany. BMC Public Health.

[31] Kinderarmut K. Fachberatung Jugendhilfeplanung. Wissen, was wirkt. Arbeitshilfe für ein wirkungsorientiertes Monitoring kommunaler Präventionsketten gegen Kinderarmut. Köln: LVR-Landesjugendamt Rheinland; 2021. Available from: https://www.lvr.de/media/wwwlvrde/jugend/jugendmter/koordinationsstellekinderarmut/dokumente_80/LVR-Broschuere_Monitoring_Praeventionsketten~1.pdf [heruntergeladen: 31.05.2021].

[32] Fröhlich-Gildhoff K, Trojan A (2018). Komplexe Gemeindeorientierte Interventionen Zur Gesundheitsförderung Bei Kindern. Evaluation am Beispiel Einer ländlichen (Ortenaukreis) und einer städtischen Region (Lenzviertel Hamburg). Bundesgesundheitsblatt Gesundheitsforschung Gesundheitsschutz.

[33] Reisig V, Jordan S, Starker A, et al. Präventionsberichterstattung – neue Impulse für die Gesundheitsberichterstattung?. Bundesgesundheitsbl. 2020;63:1118–25. 10.1007/s00103-020-03202-y. 10.1007/s00103-020-03202-y 32757021

[34] Nutbeam D (1998). Health promotion glossary. Health Promot Int.

[35] Bader B, Coenen M, Hummel J, Schoenweger P, Voss S, Jung-Sievers C (2023). Evaluation of community-based health promotion interventions in children and adolescents in high-income countries: a scoping review on strategies and methods used. BMC Public Health.

[36] Varnaccia G, Zeiher J, Lange C, Jordan S (2017). Adipositasrelevante Einflussfaktoren Im Kindesalter – Aufbau eines bevölkerungsweiten monitorings in Deutschland. J Health Monit.

[37] Niederberger M, Spranger J. Delphi Technique in Health Sciences: A Map. Front Public Health. 2020;8:457. 10.3389/fpubh.2020.00457. 10.3389/fpubh.2020.00457 PMC7536299 33072683

[38] Kuhn J, Ziese T, Prävention (2020). Präventionsberichterstattung, Methoden, Methodenprobleme. Über Prävention berichten–aber wie? Methodenprobleme Der Präventionsberichterstattung.

