# Supplementary material for: Consented indicators for the evaluation of integrated strategies of community health promotion targeting children and adolescents: results of an eDelphi
Source: BMC Public Health. 2024 Jan 22;24:252. doi: 10.1186/s12889-023-17370-4 (PMC10802006; doi:10.1186/s12889-023-17370-4)
Supplement: Supplementary file 1 — Additional file 1: Table A.1 Inclusion and exclusion criteria for single indicators’ extraction on the basis of the scoping review 1 according to the PICo (Population, Interest, Context) scheme. Table A.2 List of 40 OECD indicators to guide the participants by suggesting social and educational indicators. Table A.3 List of indicator subdomains and associated indicators after round 1. Table A.4 List of indicator subdomains after round 1 and round 2Table A.5 List of indicator subdomains and associated indicators after round 2. Table A.6 Examples of additional indicators suggested by panelists in rounds 1 and 2. [file 12889_2023_17370_MOESM1_ESM.docx]

***Additional files of:***

**Consented Indicators for the Evaluation of Integrated Strategies of Community Health Promotion targeting Children and Adolescents: Results of an eDelphi**

Myriam Robert^1 2^, Michaela Coenen^1 2^, Julia Bauer^1 2^, Stephan Voss^1 2^, Caroline Jung-Sievers^1 2^ *

^1^ Chair of Public Health and Health Services Research, Institute for Medical Information Processing, Biometry, and Epidemiology (IBE), Faculty of Medicine, LMU Munich, Munich, Germany
^2^Pettenkofer School of Public Health, Munich, Germany

*Corresponding author; Email: cjungsievers@ibe.med.uni-muenchen.de

**Table A.1** Inclusion and exclusion criteria for single indicators’ extraction on the basis of the scoping review ^1^ according to the PICo (Population, Interest, Context) scheme

**Inclusion criteria**

*Population*

- Children/adolescents aged 0-19 years (according to WHO classifications^2^)
- Mothers with their children
- Children, adolescents (aged 0-19 years) and their parents or family ^3^

*Interest*

- Health indices combining different health indicators into a composite score
- Health indicators incl. non-health sector health determinants

*Context*

- Health promotion and prevention
- Monitoring of health indicators
- Oral health

**Exclusion criteria**

*Population*

- Aged over 19 years^4^
- General population^4^ or clinical population

*Interest*

- Indicators from an area other than health^5^
- Indicators on Quality of Life^6^
- Studies with the purpose of examining quality criteria of a diagnostic instrument
- Indicators that are used for the calculation of extracted indices^7,8^
- Additional information surrounding a health indicator ^7^
- Cause of death, death reporting, death reviewing^7^

*Context*

- Clinical interventions or clinical settings
- Indicators with a clinical aim such as health care delivery or quality of care
- Association studies and predictors of health indicators
- Non-German context (foreign programs and policies)^7^

In addition, the reviewers agreed on the following:

- Vaccination status is considered as a single indicator
- Mortality rate is considered as a single indicator
- Recruitment in hospitals does not lead to exclusion if the other criteria are met
- Nutritional supplementation was included as a preventive measure

*Notes*. ^1^ Selmani A, Coenen M, Voss S, Jung-Sievers C. Health indices for the evaluation and monitoring of health in children and adolescents in prevention and health promotion: a scoping review. BMC Public Health. 2021;21(1):2309. ^2^ World Health Organization. Recognizing adolescence [Website]. Available at: https://apps.who.int/adolescent/second-decade/section2/page1/recognizing-adolescence.html [last access: 09.08.2021]. ^3^ Indicators targeting households or general population were extracted only if considered as child/mother health indicators; ^4^ For text screening only; didn’t lead to exclusion for indicators’ extraction; ^5^ Exceptions were made for indicators from non-health areas that were considered as health determinants in the publications; ^6^ For text screening: if exclusively from Quality-of-Life area; for indicators’ extraction: if explicitly named as such; ^7^ For indicators’ extraction only; ^8^ Exceptions were made for indicators that were used differently than in the index calculation (different cut-off value, different definition).

**Table A.2** List of 40 OECD indicators to guide the participants by suggesting social and educational indicators

| **Indicator (as described in OECD report)** | **Additional information from the report** |
| --- | --- |
| **Social indicators^1^** |  |
| Income inequality and percentage choosing "education" as one of their most needed supports | From the 2018 OECD Risks that Matter survey; respondents were asked which supports they would need most from government to make them feel more economically secure. They could choose from a list of nine supports, and had the option of selecting zero, one, two, or three supports. |
| Foreign-born as percentage of total populations, and percentages of recent immigrants, 15- to 64-year-olds | “Foreign-born” includes different categories of people, i.e., they can either be born in their parents’ host country to two foreign-born parents; or to mixed parentage (one foreign-born parent); be foreign-born and arrived as children; or be foreign-born and arrived as adults |
| Employment rate, percentage of the working-age population (aged 15-64), by gender | Employment is the proportion of the population aged 15-64 who are employed |
| Proportion of NEETs (not on education, employment or training) in percentage of 15-29 year-olds, by status of joblessness | Status of joblessness: unemployed or inactive |
| Proportion of inactive, unemployed or involuntary part-timers (15-64 years) in population (%), excluding youth (15-29 years) in education and not in employment | This is a broader measure of joblessness and underemployment to assess broad labor underutilization |
| Percentage of employment in shortage, by skill level | Jobs in shortage: jobs that are “hard-to-find” (in contrary to “easy-to-find” jobs) due to imbalances on the labor market. Skill level: high-skilled, medium-skilled, low-skilled |
| Percentage of persons living with less than 50% of median equalized disposable income, by gender | Disposable income is the income from work and capital after taking into account public cash transfers received and direct taxes and social security contributions paid. It excludes in-kind services provided to households by governments and private entities, consumption taxes, and imputed income flows due to home ownership. After subtracting taxes and adding cash transfers, household income provides an indication of the goods and services families can purchase on the market. Household income is adjusted for differences in the needs of households of different sizes with an equivalence scale that divides household income by the square root of household size. The adjusted income is then attributed to every person in the household |
| Percentage of persons living with less than 50% of median equalized disposable income, by age group (child/young/adult/elderly) | Equalized disposable income: see above |
| Percentage point changes in relative and “anchored” poverty rates | Indicator which measures poverty changes (in this report between 2007 and 2016) against a benchmark “anchored” to half of the median real incomes observed in 2005. |
| Working-age cash transfers paid as percentage of the working-age population, decomposed by benefit type in 2016 and total level in 2007 | Benefit types: 1) minimum income benefits, 2) unemployment assistance, 3) unemployment insurance |
| Net income while out of work in percentage of net income in work (net replacement rate, NRR), 40 years-old single | The net replacement rate (NRR) measures the fraction of net income in work that is maintained when unemployed. The indicator is separated into NRR referring to an initial employment phase (in second month of benefit following any waiting period) and NRR referring to a long-term unemployment phase NRR (in 60th month of benefit receipt) |
| Net income level provided by cash minimum income benefit (MIB), single person, with and without housing benefit (HB), in percentage of median household income | Median household income: see above In the report, the minimum income benefit and the housing benefit are compared to poverty thresholds (50 and 60%) |
| Proportion of public cash transfers received by working-age individuals in low- and high-income groups | The low- and high-income groups here are the poorest and richest quintiles (the poorest 20% and the richest 20%) |
| Housing cost burden: proportion of population in the bottom quintile of the income distribution spending more than 40% of disposable income on mortgage and rent, by tenure, percentages | This indicator distinguishes between private rent (in for-profit housing market at market-rates), subsidized rent (social rental housing at sub-market prices) and owner with mortgage |
| Proportion of children (aged 0-17) living in overcrowded households (in European OECD countries), by income group, percentages | Overcrowded household: not having at its disposal a minimum number of rooms equal to: one room for the household; one room per adult couple in the household; one room for each single person aged 18 and over; one room per pair of single persons of the same sex between 12 and 17 years of age; one room for each single person between 12 and 17 years of age and not included in the previous category; one room per pair of children under 12 years of age (EU-agreed definition from Eurostat) |
| Gap in life expectancy at age 30 between tertiary and below upper secondary education, by gender | Life expectancy at birth measures how long, on average, people would live based on a given set of age-specific death rates |
| Life expectancy at birth in years, and health spending per capita in USD PPP (Purchasing Power Parities) | Heath spending measures the final consumption of health goods and services, including spending by both public and private sources on medical services and goods, as well as public health and prevention programs and administration, but excluding spending on capital formation (investments in infrastructure, machinery and equipment, as well as software and databases). Use of USD: for comparison purposes across countries. PPP: Adjustment to take account of the different purchasing power of the national currencies using Purchasing Power Parities (PPPs) exchange rates |
| Age-standardized suicide rate per 100 000 population by gender; suicide rate per 100 000 population, by age-group and gender | In the 2019 OECD report, the rates were age-standardized to the 2010 OECD population to remove variations arising from differences in age structures across countries and over time |
| Trends in age-standardized suicide rate per 100 000 population | In the 2019 OECD report, trends are presented between 1970 and 201; Age-standardized: see above |
| Average points of life satisfaction on a scale from 0 to 10; average points of life satisfaction on a scale from 0 to 10, by socio-demographic group | The 2019 OECD report presents the OECD average, the minimum and the maximum values of life satisfaction and disaggregates data by gender, age group, employment type (full-time job vs. other), education (tertiary completed vs. less than tertiary), income quintile and geography (urban/rural) |
| Positive versus negative experience index | The positive experience index averages country responses to five questions about whether the respondent experienced a lot of enjoyment, smiled or laughed a lot, felt well-rested and learned or did something interesting the day before the interview. The negative experience index averages country responses to five questions about whether the respondent experienced a lot of physical pain, worry, stress, sadness and anger. The index scores are the mean of all valid affirmative responses to these items multiplied by 100 |
| Percentage of people reporting having confidence in national government, by household income | Data on confidence is based on binary questions: Do you have confidence in each of the following: in the national government, in financial institutions or banks, in the judicial system and courts, in the local police force, in the military. Data on corruption perception is based on the binary question: “Is corruption widespread throughout the government in this country, or not?” |
| Trend in percentage of people reporting having confidence in specific institution (national government, financial institutions, judicial system, local police, military) | Data on confidence: see above |
| Internet users by age, as a percentage of the population in each age group | Internet users are defined for a recall period of three months |
| Daily time spent on the Internet by young people and all individuals | Data on daily time spent on the Internet: from the European Social Survey (ESS; face-to-face interviews with newly selected, cross-sectional samples every two years) |
| **Educational indicators^2^** |  |
| Educational attainment of 25-64 year-olds, 25-34 year-olds und 55-64 year-olds by program orientation | Proportion of adults with a given (vocational/general) qualification as their highest educational attainment. Program orientation: general, vocational or both |
|  |  |
| Trends in educational attainment of 25-34 year-olds by gender | Proportion of adults with a given qualification as their highest educational attainment. In the OECD reports, indicators are presented for 2009 and 2019 |
| Percentage of 18-24 year-olds in education or not in education by labor-market status | Labor-market status: unemployed, inactive, employed |
|  |  |
| Trends in the percentage of young adults in education or not in education by age group and labor-market status | Age groups: 20-24 year-olds, 15-29 year-olds  Labor-market status: unemployed, inactive, employed.  Not in education: including NEET defined as neither employed nor in education/training |
| Young adults with upper secondary education who are in education or not in education; employed or unemployed, by years since graduation | 15-34 years-olds by the time of graduation  By time since graduation and labor-market status; not in education including NEET defined as neither employed nor in education/training |
| Level of earnings relative to median earnings by educational attainment | Including part- and full-time workers. Relation to median: at or below half of the median; more than half the median but at or below the median; more than the median but at or below 1,5 times; more than twice the median |
| Women’s earnings as a percentage of men’s earnings by educational attainment and age group | Average earnings of adults with income from employment (full-time full-year workers) |
| Enrolment rates of 15-19 year-old students and 20-24 year-old students in secondary education und tertiary education by level of education; enrolment rates of students aged 25 years and older in secondary education und tertiary education by level of education | Students enrolled in full-time and part-time programs in both public and private institutions |
| Distribution of entrants to upper secondary education, by program orientation at entrance and outcomes after the theoretical duration and after the theoretical duration plus two years | Program orientation: general, vocational or both |
| Entry rate and profile of first-time entrants into tertiary education | Characteristics: proportion of female first-time entrants/below the age of 25 years//internationals; average age; proportion of first-time entrants by level of education (short-cycle tertiary/Bachelor’s or equivalent/Master’s or equivalent) |
| Entry rate and profile of entrants into short-cycle tertiary education | Characteristics: proportion of female first-time entrants/below the age of 25 years//internationals; average age; proportion of first-time entrants by field |
| Profile of students enrolled in vocational education from lower secondary to short-cycle tertiary education by type of program, age and gender | Characteristics: proportion of students in lower/upper/secondary education/ post-secondary non-tertiary education/short-cycle tertiary education; proportion of students in vocational lower secondary education (proportion of students enrolled in vocational education and training as a % of all students enrolled at this level; % of female; % aged 16 and older); proportion of students in vocational upper secondary education (proportion of students enrolled in vocational education and training as a % of all students enrolled at this level; % of female; % aged 20/25 and older) |
| Pathways between upper secondary or post-secondary non-tertiary education and higher levels of education, by type of program and program orientation | Average age of enrolment in upper secondary education; proportion of students with insufficient (or partial level of) completion without access to tertiary education/with full level of completion without access to tertiary education/with full level of completion with direct access to tertiary education by program orientation: general, vocational or both |
| Tasks of teachers by level of education  Other responsibilities of teachers by level of education | For lower secondary, general programs. By type: mandatory/school requirement/voluntary. Tasks: teaching/individual planning or preparation of lessons either at school or elsewhere; marking/correcting of students’ work; general administrative work (including communication, paperwork and other clerical duties undertaken as part of the job); communication and cooperation with parents or guardians; supervisions of students during breaks; team work and dialogue with colleagues at school or elsewhere |
|  | For lower secondary, general programs. By type: mandatory/school requirement/voluntary. Work requirements: participation in school or other management in addition to teaching duties (e.g., serving as head of department or coordinator of teachers; teaching more classes or hours than required by full-time contract; student counselling (including student supervision, virtual counselling, career guidance, and delinquency prevention); engaging in extracurricular activities (e.g., homework clubs, sports and drama clubs, summer school); special tasks (e.g., training student teachers, guidance counselling); class teacher/form teacher; participation in mentoring programs and/or supporting new teachers in induction programs; participation in professional development activities |

*Notes*. ^1^ OECD. Society at a Glance 2019: OECD Social Indicators. Paris: OECD Publishing; 2019^. 2^ OECD. Bildung auf einen Blick 2020: OECD-Indikatoren. Bielefeld: wbv Media; 2020.
The eDelphi participants had access to a guide (in English and German) with the indicators and further information mentioned above. To prevent misunderstandings, additional definitions were also included.

**Table A.3** List of indicator subdomains and associated indicators after round 1

| **Subdomain** **(consensus level on relevance)** | | **Indicators** | **Consensus level on relevance^1^** | **N** | **Median** | |
| --- | --- | --- | --- | --- | --- | --- |
| **Socioeconomic factors (100%)** | | |  |  | |  |
|  | Proportion of early school leavers | | 91% | 50/55 | | 5 |
|  | Child poverty rate | | 89% | 49/55 | | 5 |
|  | Perceived social support at the individual level | | 87% | 48/55 | | 5 |
|  | Percentage of women and children with inadequate social support | | 87% | 48/55 | | 5 |
|  | Children with supportive neighborhood | | 78% | 43/55 | | 4 |
|  | *Prevalence of adverse childhood experiences among children* | | 69% | 38/55 | | 4 |
| **Health education (98%)** | | |  |  | |  |
|  | Preventive oral health programs in kindergartens | | 78% | 42/54 | | 4 |
| **Nutrition and physical activity (95%)** | | |  |  | |  |
|  | Physical activity | | 87% | 46/53 | | 4 |
|  | Nutritional behavior | | 85% | 45/53 | | 4 |
|  | Physical activity as organized physical activity | | 79% | 42/53 | | 4 |
|  | Physical inactivity | | 77% | 41/53 | | 5 |
|  | *Sedentary behavior* | | 72% | 38/53 | | 4 |
|  | *Fruit and vegetables consumption* | | 60% | 32/53 | | 4 |
|  | *(In)adequacy of dietary intake* | | 53% | 28/53 | | 4 |
|  | *Current fruit consumption* | | 53% | 28/53 | | 4 |
|  | *Vegetables consumption* | | 53% | 28/53 | | 4 |
| **Health status as specific physical, mental health conditions (91%)** | | |  |  | |  |
|  | Children with developmental delay | | 84% | 43/51 | | 5 |
|  | Refusal to attend school | | 82% | 42/51 | | 5 |
|  | Emotional distress | | 80% | 41/51 | | 4 |
|  | Depression | | 77% | 39/51 | | 4 |
|  | Subjective health complaints | | 77% | 39/51 | | 4 |
|  | *Eating disorder* | | 75% | 38/51 | | 4 |
|  | *Psychosomatic complaints* | | 71% | 36/51 | | 4 |
|  | *Sleep disorder* | | 67% | 34/51 | | 4 |
|  | *Asthma* | | 47% | 24/51 | | 4 |
|  | *Allergy and/or asthma and/or eczema* | | 47% | 24/51 | | 4 |
|  | *Diabetes* | | 47% | 24/51 | | 3,5 |
|  | *Number of mentally unhealthy days in the past month* | | 45% | 23/51 | | 4 |
|  | *Cardiovascular disease* | | 43% | 22/51 | | 3 |
|  | *Health conditions reported in children’s medical health history* | | 39% | 20/51 | | 3 |
|  | *Weekly health complaints* | | 35% | 18/51 | | 3 |
| **Drug related behavior (91%)** | | |  |  | |  |
|  | Illicit drug dependence | | 90% | 46/51 | | 4 |
|  | Extreme/harmful alcohol consumption | | 82% | 42/51 | | 4 |
|  | First alcohol consumption before age 13 years | | 78% | 40/51 | | 4 |
|  | Current alcohol consumption | | 77% | 39/51 | | 4 |
|  | Alcohol dependence | | 77% | 39/51 | | 4 |
|  | *First cigarette smoking before age 13 years* | | 75% | 38/51 | | 4 |
|  | *Current overall tobacco use* | | 73% | 37/51 | | 4 |
|  | *Tobacco dependence* | | 69% | 35/51 | | 4 |
|  | *Total alcohol consumption* | | 69% | 35/51 | | 4 |
|  | *Smokeless tobacco consumption* | | 37% | 19/51 | | 3 |
|  | *Children who have ever smoked cigarettes* | | 27% | 14/51 | | 3 |
| **Overall health status (88%)** | | |  |  | |  |
|  | *Perceived overall health status* | | 69% | 34/49 | | 4 |
| **Oral health (86%)** | | |  |  | |  |
|  | DMFT (decayed, missing, filled, tooth) index | | 83% | 40/48 | | 4 |
| **Health behavior as a combination of multiple factors (86%)** | | |  |  | |  |
|  | HLI (Healthy Lifestyle Index) | | 77% | 37/48 | | 4 |
| **Exposure to drugs and violence (84%)** | | |  |  | |  |
|  | Number of children reported abused or neglected | | 87% | 40/46 | | 5 |
|  | Exposure to physical violence in the community | | 87% | 40/46 | | 5 |
|  | Substantiated child maltreatment including experience of physical abuse, neglect or deprivation of necessities, medical neglect, sexual abuse, psychological or emotional maltreatment | | 83% | 38/46 | | 5 |
|  | *Children in smoking household* | | 74% | 34/46 | | 4 |
|  | *Intimate partner violence, injury, physical or sexual abuse* | | 67% | 31/46 | | 4 |
|  | *Children who had ever been physically forced to have sexual intercourse when they did not want* | | 59% | 27/46 | | 4 |
| **Family factors (80%)** | | |  |  | |  |
|  | Smoking during pregnancy | | 80% | 35/44 | | 4 |
|  | *Adult overweight or obesity* | | 70% | 31/44 | | 4 |
|  | *Percentage of adults with diagnosed diabetes* | | 36% | 16/44 | | 3 |
|  | *HPV (human papillomavirus) immunization among young adults aged 18–26 years* | | 21% | 9/44 | | 3 |
| **Functional health status (79%)** | | |  |  | |  |
|  | Activity limitation | | 84% | 37/44 | | 4 |
|  | *HUI (Health Utilities Index)* | | 68% | 30/44 | | 4 |
|  | *Motor coordination* | | 43% | 19/44 | | 3 |
|  | *Muscle flexibility* | | 39% | 17/44 | | 3 |
|  | *Fitness testing measures* | | 30% | 13/44 | | 3 |
|  | *Muscle strength* | | 23% | 10/44 | | 2 |
|  | *Jumping distance* | | 16% | 7/44 | | 2 |
| ***Immunization (73%)*** | | |  |  | |  |
|  | Full immunization | | 85% | 34/40 | | 4 |
|  | Measles immunization | | 75% | 30/40 | | 4 |
|  | *Presence of immunization card* | | 73% | 29/40 | | 4 |
|  | *DPT immunization (diphtheria, pertussis, tetanus)* | | 58% | 23/40 | | 4 |
|  | *IPV immunization (poliovirus)* | | 50% | 20/40 | | 4 |
|  | *BCG immunization (tuberculosis)* | | 40% | 16/40 | | 4 |
|  | *HPV immunization (human papillomavirus) among adolescents* | | 38% | 15/40 | | 3 |
|  | *Hib immunization (Haemophilus influenzae type B)* | | 28% | 11/40 | | 2 |
| ***Combination of multiple factors (71%)*** | | |  |  | |  |
|  | *Health indicators for Swedish Children* | | 62% | 24/39 | | 4 |
| ***Anthropometric*** ***factors (68%)*** | | |  |  | |  |
|  | *Obesity* | | 74% | 28/38 | | 5 |
|  | *Underweight* | | 74% | 28/38 | | 5 |
|  | *Overweight (with obesity)* | | 63% | 24/38 | | 4 |
|  | *Overweight (without obesity)* | | 63% | 24/38 | | 4 |
|  | *BMI percentiles for age and sex* | | 61% | 23/38 | | 4 |
|  | *BMI z-scores for age and sex* | | 55% | 21/38 | | 4 |
|  | *BMI* | | 45% | 17/38 | | 3 |
|  | *Weight-for-height z-scores by age and sex* | | 42% | 16/38 | | 4 |
| ***Teen births (68%)*** | | |  |  | |  |
|  | *Adolescent mothers* | | 74% | 28/38 | | 4 |
| ***Sleep behavior (64%)*** | | |  |  | |  |
|  | Sleep duration | | 81% | 29/36 | | 4 |
| ***Breastfeeding (62%)*** | | |  |  | |  |
|  | *Exclusive breastfeeding rate for 6 months* | | 56% | 19/34 | | 4 |
| ***Child mortality (54%)*** | | |  |  | |  |
|  | Under-age-1-mortality | | 83% | 25/30 | | 4 |
|  | Under-age-5 mortality | | 83% | 25/30 | | 4 |
|  | *Still births* | | 63% | 19/30 | | 4 |
|  | *Perinatal mortality* | | 50% | 15/30 | | 4 |
|  | *Neonatal mortality* | | 43% | 13/30 | | 3 |
|  | *Late neonatal mortality* | | 43% | 13/30 | | 3 |
|  | *Postneonatal mortality* | | 43% | 13/30 | | 3,5 |
|  | *Early neonatal mortality* | | 40% | 12/30 | | 3 |
|  | *Postneonatal mortality of term infants weighing ≤ 2500g at birth* | | 33% | 10/30 | | 3 |
| ***Contraception (47%)*** | | |  |  | |  |
|  | *Current condom use* | | 58% | 15/26 | | 4 |
| ***Laboratory parameters (34%)*** | | |  |  | |  |
|  | *Mean arterial pressure* | | 74% | 14/19 | | 4 |
|  | *Blood glucose and insulin levels* | | 58% | 11/19 | | 4 |

*Notes*. Subdomains and indicators shaded in green reached a consensus on relevance (rated by ≥75% as *relevant* or *very relevant*). Grey and italic items did not reach any consensus either on relevance or on irrelevance. ^1^ Percentage of panelists who rated the subdomain or indicator as *relevant* or *very relevant*, incl. *not specified* responses.

**Table A.4** List of indicator subdomains after round 1 and round 2

| \| **ROUND 1** \| \| \| --- \| --- \| \| **Relevant subdomains** (consensus on relevance: ≥75% of the panelists rated the subdomain as *relevant)* \| **Consensus level on relevance^1^** \| \| Socioeconomic factors \| 100,0% (56/56) \| \| Health education \| 98,2% (54/55) \| \| Nutrition and physical activity \| 94,6% (53/56) \| \| Health status as specific physical, mental health conditions \| 91,1% (51/56) \| \| Drug related behavior \| 91,1% (51/56) \| \| Overall health status \| 87,5% (49/56) \| \| [Oral](https://expertsurveytest.limequery.com/upload/surveys/221257/files/Dental%20health.pdf) health \| 85,7% (48/56) \| \| Health behavior as a combination of multiple factors \| 85,7% (48/56) \| \| Exposure to drugs and violence \| 83,6% (46/55) \| \| [Family](https://expertsurveytest.limequery.com/upload/surveys/221257/files/Familial.pdf) factors \| 80,0% (44/55) \| \| Functional health status \| 78,6% (44/56) \| \| **Irrelevant subdomains** (consensus of irrelevance: ≥75% of the panelists rated the subdomain as *not relevant)* \| **-** \| \| *None* \| - \| \| **Remaining subdomains**  (not reaching consensus either on relevance or on irrelevance) \| **Consensus level on relevance** \| \| Immunization \| 72,7% (40/55) \| \| [Combination](https://expertsurveytest.limequery.com/upload/surveys/221257/files/Combination%20Swedish%20indic.pdf) of multiple factors \| 70,9% (39/55) \| \| Anthropometric factors \| 67,9% (38/56) \| \| Teen births \| 67,9% (38/56) \| \| [Sleep](https://expertsurveytest.limequery.com/upload/surveys/221257/files/Sleep.pdf) behavior \| 64,3% (36/56) \| \| Breastfeeding \| 61,8% (34/55) \| \| [Child mortality](https://expertsurveytest.limequery.com/upload/surveys/221257/files/10_09_Mortalit%C3%A4t.pdf) \| 53,6% (30/56) \| \| Contraception \| 47,3% (26/55) \| \| [Laboratory parameters](https://expertsurveytest.limequery.com/upload/surveys/221257/files/Vital%20labor.pdf) \| 33,9% (19/56) \| | \| **ROUND 2** \| \| \| \| --- \| --- \| --- \| \| **Relevant subdomains** (consensus on relevance: ≥75% of the panelists rated the subdomain as *relevant)* \| \| **Consensus level on relevance** \| \| Health education \| \| 100,0% (47/47) \| \| Socioeconomic factors \| \| 100,0% (47/47) \| \| [Nutrition and physical activity](https://expertsurveytest.limequery.com/upload/surveys/221257/files/Activitiesnutrition.pdf) \| \| 100,0% (47/47) \| \| Oral health \| \| 97,9% (47/48) \| \| Overall health status \| \| 93,8% (45/48) \| \| Health status as specific physical, mental health conditions \| \| 87,8% (43/49) \| \| Drug related behavior \| \| 87,5% (42/48) \| \| Exposure to drugs and violence \| \| 83,3% (40/48) \| \| Family factors \| \| 78,7% (37/47) \| \| **Irrelevant subdomains** (consensus on irrelevance: ≥75% of the panelists rated the subdomain as *not relevant)* \| \| **Consensus level on irrelevance** \| \| Vital and laboratory parameters**^2^** \| 83,3% *not relevant* (40/48)  (16,7% *relevant* (8/48)) \| \| \| **Remaining subdomains**  (not reaching consensus either on relevance or on irrelevance) \| \| **Consensus level on relevance** \| \| Health behavior as a combination of multiple factors \| \| 74,5% (35/47) \| \| Immunization \| \| 70,2% (33/47) \| \| [Combination](https://expertsurveytest.limequery.com/upload/surveys/221257/files/Combination%20Swedish%20indic.pdf) of multiple factors \| \| 70,2% (33/47) \| \| [Sleep](https://expertsurveytest.limequery.com/upload/surveys/221257/files/Sleep.pdf) behavior \| \| 68,1% (32/47) \| \| Anthropometric factors \| \| 66,7% (32/48) \| \| Functional health status \| \| 64,6% (31/48) \| \| Breastfeeding \| \| 59,6% (28/47) \| \| Teen births \| \| 58,3% (28/48) \| \| Contraception \| \| 38,3% (18/47) \| \| [Child mortality](https://expertsurveytest.limequery.com/upload/surveys/221257/files/10_09_Mortalit%C3%A4t.pdf) \| \| 33,3% (16/48) \| |
| --- | --- | --- | --- | --- | --- | --- | --- | --- | --- | --- | --- | --- | --- | --- | --- | --- | --- | --- | --- | --- | --- | --- | --- | --- | --- | --- | --- | --- | --- | --- | --- | --- | --- | --- | --- | --- | --- | --- | --- | --- | --- | --- | --- | --- | --- | --- | --- | --- | --- | --- | --- | --- | --- | --- | --- | --- | --- | --- | --- | --- | --- | --- | --- | --- | --- | --- | --- | --- | --- | --- | --- | --- | --- | --- | --- | --- | --- | --- | --- | --- | --- | --- | --- | --- | --- | --- | --- | --- | --- | --- | --- | --- | --- | --- | --- | --- | --- | --- | --- | --- | --- | --- | --- | --- | --- | --- | --- | --- | --- | --- | --- | --- | --- | --- | --- | --- | --- | --- | --- | --- | --- | --- | --- |

*Notes*. Subdomains shaded in green reached a consensus on relevance (rated by ≥75% as *relevant*). Subdomains shaded in red reached a consensus on irrelevance (rated by ≥75% as *not* *relevant*). Grey items did not reach any consensus either on relevance or on irrelevance. ^1^ Percentage of panelists who rated the subdomain as *relevant*, incl. *not specified* responses. ^2^ The subdomain’s formulation was changed in round 2.

**Table A.5** List of indicator subdomains and associated indicators after round 2

| **Subdomain (consensus level on relevance)** | | | | **Indicators** | | **Consensus level on relevance^1^** | | | **N** | **Median** | | | | | | | | |  |
| --- | --- | --- | --- | --- | --- | --- | --- | --- | --- | --- | --- | --- | --- | --- | --- | --- | --- | --- | --- |
| **Socioeconomic factors (100%)** | | | | |  | | | | | | | | | | |  | | |  |
|  | | Proportion of early school leavers | | | | 89% | | 42/47 | | | | 5 | | | | | | |  |
|  | | Percentage of women and children with inadequate social support | | | | 89% | | 42/47 | | | | 5 | | | | | | |  |
|  | | Perceived social support at the individual level | | | | 89% | | 42/47 | | | | 5 | | | | | | |  |
|  | | Child poverty rate | | | | 87% | | 41/47 | | | | 5 | | | | | | |  |
|  | | Children with supportive neighborhood | | | | 85% | | 40/47 | | | | 5 | | | | | | |  |
|  | | | *Prevalence of adverse childhood experiences among children* | | | 70% | | 33/47 | | | 5 | | | | | | | |  |
| **Nutrition and physical activity (100%)** | | | | |  | | | | | | | | | | | | | |  |
|  | | Physical activity | | | | 96% | | 45/47 | | | | 4 | | | | | | |  |
|  | | Physical inactivity | | | | 94% | | 44/47 | | | | 5 | | | | | | |  |
|  | | Physical activity as organized physical activity | | | | 92% | | 43/47 | | | | 4 | | | | | | |  |
|  | | Sedentary behavior | | | | 89% | | 42/47 | | | | 5 | | | | | | |  |
|  | | Nutritional behavior | | | | 89% | | 42/47 | | | | 4 | | | | | | |  |
|  | *(In)adequacy of dietary intake* | | | | | 70% | | 33/47 | | | | | 4 | | | | | |  |
|  | *Fruit and vegetables consumption* | | | | | 68% | | 32/47 | | | | | 4 | | | | | |  |
|  | *Vegetables consumption* | | | | | 53% | | 25/47 | | | | | 4 | | | | | |  |
|  | *Current fruit consumption* | | | | | 51% | | 24/47 | | | | | 4 | | | | | |  |
| **Health education (100%)** | | | | | |  | |  | | | |  | | | | | | |  |
|  | | Preventive oral health programs in kindergartens | | | | 87% | | 41/47 | | | | 4 | | | | | | |  |
| **Oral health (98%)** | | | | | |  | |  | | | |  | | | | | | |  |
|  | | DMFT (decayed, missing, filled, tooth) index | | | | 87% | | 41/47 | | | | 4 | | | | | | |  |
| **Overall health status (94%)** | | | | | | | |  | | | |  | | | | | | |  |
|  | | Perceived overall health status | | | | 80% | | 36/45 | | | | 4 | | | | | | |  |
| **Health status as specific physical, mental health conditions (88%)** | | | | |  | | | | | | | | | | | | | |  |
|  | | Children with developmental delay | | | | 100% | | 42/42 | | | | 5 | | | | | | |  |
|  | | Refusal to attend school | | | | 95% | | 40/42 | | | | 5 | | | | | | |  |
|  | | Depression | | | | 88% | | 37/42 | | | | 5 | | | | | | |  |
|  | | Emotional distress | | | | 88% | | 37/42 | | | | 4 | | | | | | |  |
|  | | Subjective health complaints | | | | 83% | | 35/42 | | | | 4 | | | | | | |  |
|  | | Eating disorder | | | | 83% | | 35/42 | | | | 4 | | | | | | |  |
|  | *Psychosomatic complaints* | | | | | 69% | | 29/42 | | | | | 4 | | | | | |  |
|  | *Sleep disorder* | | | | | 69% | | 29/42 | | | | | 4 | | | | | |  |
|  | *Allergy and/or asthma and/or eczema* | | | | | 48% | | 20/42 | | | | | 3,5 | | | | | |  |
|  | *Number of mentally unhealthy days in the past month* | | | | | 43% | | 18/42 | | | | | 3 | | | | | |  |
|  | *Diabetes* | | | | | 43% | | 18/42 | | | | | 3 | | | | | |  |
|  | *Cardiovascular disease* | | | | | 43% | | 18/42 | | | | | 3 | | | | | |  |
|  | *Asthma* | | | | | 41% | | 17/42 | | | | | 3 | | | | | |  |
|  | *Health conditions reported in children’s medical health history* | | | | | 24% | | 10/42 | | | | | 3 | | | | | |  |
|  | *Weekly health complaints* | | | | | 19% | | 08/42 | | | | | 2 | | | | | |  |
| **Drug related behavior (88%)** | | | | |  | | | | | | | | | | | | | |  |
|  | | Current alcohol consumption | | | | 100% | | 42/42 | | | | 5 | | | | | | |  |
|  | | Extreme/harmful alcohol consumption | | | | 98% | | 41/42 | | | | 5 | | | | | | |  |
|  | | Illicit drug dependence | | | | 95% | | 40/42 | | | | 4 | | | | | | |  |
|  | | Current overall tobacco use | | | | 93% | | 39/42 | | | | 4 | | | | | | |  |
|  | | Alcohol dependence | | | | 88% | | 37/42 | | | | 4 | | | | | | |  |
|  | | First cigarette smoking before age 13 years | | | | 88% | | 37/42 | | | | 4 | | | | | | |  |
|  | | Total alcohol consumption | | | | 88% | | 37/42 | | | | 5 | | | | | | |  |
|  | | First alcohol consumption before age 13 years | | | | 86% | | 36/42 | | | | 4 | | | | | | |  |
|  | | Tobacco dependence | | | | 81% | | 34/42 | | | | 4 | | | | | | |  |
|  | *Smokeless tobacco consumption* | | | | | 74% | | 31/42 | | | | | 4 | | | | | |  |
|  | *Children who have ever smoked cigarettes* | | | | | 24% | | 10/42 | | | | | 2,5 | | | | | |  |
| **Exposure to drugs and violence (83%)** | | | | | |  | |  | | | |  | | | | | | |  |
|  | | Children in smoking household | | | | 85% | | 34/40 | | | | 5 | | | | | | |  |
|  | | Number of children reported abused or neglected | | | | 85% | | 34/40 | | | | 5 | | | | | | |  |
|  | | Substantiated child maltreatment including experience of physical abuse, neglect or deprivation of necessities, medical neglect, sexual abuse, psychological or emotional maltreatment | | | | 85% | | 34/40 | | | | 5 | | | | | | |  |
|  | | Exposure to physical violence in the community | | | | 83% | | 33/40 | | | | 5 | | | | | | |  |
|  | | Intimate partner violence, injury, physical or sexual abuse | | | | 80% | | 32/40 | | | | 5 | | | | | | |  |
|  | | Children who had ever been physically forced to have sexual intercourse when they did not want | | | | 78% | | 31/40 | | | | 5 | | | | | | |  |
| **Family factors (79%)** | | | | |  | | | | | | | | | | | | | |  |
|  | | Smoking during pregnancy | | | | 87% | | 32/37 | | | | 5 | | | | | | |  |
|  | | Adult overweight or obesity | | | | 78% | | 29/37 | | | | 4 | | | | | | |  |
|  | *Percentage of adults with diagnosed diabetes* | | | | | 46% | | 17/37 | | | | | 3 | | | | | |  |
|  | *HPV (human papillomavirus) immunization among young adults aged 18–26 years* | | | | | 14% | | 05/37 | | | | | 2 | | | | | |  |
| ***Health behavior as a combination of multiple factors (74%)*** | | | | | |  | |  | | | |  | | | | | | |  |
|  | | HLI (Healthy Lifestyle Index) | | | | 89% | | 31/35 | | | | 4 | | | | | | |  |
| ***Immunization (70%)*** | | | | |  | | | | | | | | | | | | | |  |
|  | Measles immunization | | | | | 88% | 29/33 | | | | | | | 4 | | |  |  |  |
|  | Full immunization | | | | | 85% | 28/33 | | | | | | | 4 | | |  |  |  |
|  | Presence of immunization card | | | | | 76% | 25/33 | | | | | | | 4 | | |  |  |  |
|  | *DPT immunization* | | | | | 73% | | 24/33 | | | | | 4 | | | | | |  |
|  | *IPV immunization (poliovirus)* | | | | | 64% | | 21/33 | | | | | 4 | | | | | |  |
|  | *BCG immunization (tuberculosis)* | | | | | 33% | | 11/33 | | | | | 3 | | | | | |  |
|  | *Hib immunization (Haemophilus influenzae type B)* | | | | | 30% | | 10/33 | | | | | 3 | | | | | |  |
|  | *HPV immunization (human papillomavirus) among adolescents* | | | | | 27% | | 09/33 | | | | | 3 | | | | | |  |
| ***Combination of multiple factors (70%)*** | | | | |  | | | | | | | | | | | | | |  |
|  | *Health indicators for Swedish Children* | | | | | 61% | | 20/33 | | | | | 4 | | | | | |  |
| ***Sleep behavior (68%)*** | | | | | |  |  | | | | | | | |  | | | |  |
|  | Sleep duration | | | | | 84% | 27/32 | | | | | | | | 4 | | | |  |
| ***Anthropometric*** ***factors (67%)*** | | | | |  | | | | | | | | | | | | | |  |
|  | Obesity | | | | | 90% | 29/32 | | | | | | | | 5 | | | |  |
|  | Overweight (with obesity) | | | | | 90% | 29/32 | | | | | | | | 5 | | | |  |
|  | Overweight (without obesity) | | | | | 88% | 28/32 | | | | | | | | 5 | | | |  |
|  | Underweight | | | | | 88% | 28/32 | | | | | | | | 5 | | | |  |
|  | BMI z-scores for age and sex | | | | | 81% | 26/32 | | | | | | | | 4 | | | |  |
|  | BMI percentiles for age and sex | | | | | 78% | 25/32 | | | | | | | | 4 | | | |  |
|  | *Weight-for-height z-scores by age and sex* | | | | | 72% | | 23/32 | | | | | 4 | | | | | |  |
|  | *BMI* | | | | | 59% | | 19/32 | | | | | 4 | | | | | |  |
| ***Functional health status (65%)*** | | | | |  | | | | | | | | | | | | | |  |
|  | Activity limitation | | | | | 97% | 30/31 | | | | | | | | 4 | | | | |
|  | HUI (Health Utilities Index) | | | | | 84% | 26/31 | | | | | | | | 4 | | | | |
|  | *Motor coordination* | | | | | 71% | 22/31 | | | | | | | | 4 | | | | |
|  | *Motor coordination* | | | | | 71% | | 22/31 | | | | | 4 | | | | |  |  |
|  | *Muscle flexibility* | | | | | 52% | | 16/31 | | | | | 4 | | | | |  |  |
|  | *Fitness testing measures* | | | | | 26% | | 08/31 | | | | | 3 | | | | |  |  |
|  | *Muscle strength* | | | | | 16% | | 05/31 | | | | | 2 | | | | |  |  |
|  | *Jumping distance* | | | | | 10% | | 03/31 | | | | | 2 | | | | |  |  |
| ***Breastfeeding (60%)*** | | | | |  | | | | | | | | | | | | | |  |
|  | *Exclusive breastfeeding rate for 6 months* | | | | | 54% | | 15/28 | | | | | 4 | | | | | |  |
| ***Teen births (58%)*** | | | | | |  |  | | | | | | | |  | | | |  |
|  | Adolescent mothers | | | | | 86% | 24/28 | | | | | | | | 4 | | | |  |
| ***Contraception (38%)*** | | | | |  | | | | | | | | | | | | | |  |
|  | *Current condom use* | | | | | 44% | | 08/18 | | | | | 3 | | | | | |  |
| ***Child mortality (33%)*** | | | | |  | | | | | | | | | | | | | |  |
|  | Under-age-1-mortality | | | | | 94% | | 15/16 | | | | | 4 | | | | | |  |
|  | Under-age-5 mortality | | | | | 88% | | 14/16 | | | | | 4 | | | | | |  |
|  | Neonatal mortality | | | | | 81% | | 13/16 | | | | | 4 | | | | | |  |
|  | Late neonatal mortality | | | | | 75% | | 12/16 | | | | | 4 | | | | | |  |
|  | *Early neonatal mortality* | | | | | 69% | | 11/16 | | | | | 4 | | | | | |  |
|  | *Postneonatal mortality* | | | | | 69% | | 11/16 | | | | | 4 | | | | | |  |
|  | *Perinatal mortality* | | | | | 69% | | 11/16 | | | | | 4 | | | | | |  |
|  | *Still births* | | | | | 63% | | 10/16 | | | | | 4 | | | | | |  |
|  | *Postneonatal mortality of term infants weighing ≤ 2500g at birth* | | | | | 50% | | 08/16 | | | | | 4 | | | | | |  |
| **Vital and laboratory parameters (17%^2^)** | | | | |  | | | | | | | | | | | | | |  |
|  | Mean arterial pressure | | | | | 75% | | 06/08 | | | | | 4 | | | | | |  |
|  | *Blood glucose and insulin levels* | | | | | 63% | | 05/08 | | | | | 4 | | | | | |  |

*Notes.* Subdomains and indicators shaded in green reached a consensus on relevance (rated by ≥75% as *relevant* or *very relevant*). Subdomains and indicators shaded in red reached a consensus on irrelevance (rated by ≥75% as *not* *relevant* or *little relevant*). Grey and italic items did not reach any consensus either on relevance or on irrelevance. ^1^ Percentage of panelists who rated the subdomain or indicator as *relevant* or *very relevant*, incl. *not specified* responses. ^2^ Indicator subdomain rated as *not relevant* by 83% of the panelists, thereby reaching consensus on irrelevance. The subdomain’s formulation was changed in round 2.

**Table A.6** Examples of additional indicators suggested by panelists in rounds 1 and 2

| **Topic** | **Examples** |
| --- | --- |
| **General demographic data** | Mothers’ age, population distribution |
| **Family structure** | Single parent households, number of siblings |
| **Employment situation** | Parents’ employment, youth employment |
| **Income & poverty** | Household income, child poverty rate, socioeconomic status |
| **Social benefits** | Children in households drawing social benefits such as cash transfers or support institutions |
| **Housing** | Children in overcrowded households, financial burden through housing |
| **Migration** | Native language, immigration background |
| **Preschool** | Proportion of preschoolers, preschool duration, availability of kindergarten services |
| **Educational degree (teenagers, parents)** | High-school graduated teenagers, students who interrupted school, parents’ highest educational degree |
| **School education** | School year repeats, switches from school to school |
| **Social participation, access to education and exercise** | Availability of after-school activities (in sports, music, culture), memberships in after-school programs, availability and usage of school support programs |
| **Municipal services and environment** | Social/medical/health promoting institutions and related indicators, structural changes caused by health promotion intervention, availability of (close) social networks, social support |
| **Living environment** | Food environment, availability of green spaces or playing areas, public transportation’s accessibility |
| **Networks** | Municipal network’s quality, structural changes caused by health promotion (prevention chains) networks |
| **Quality of life** | Health-related quality of life, self-perceived well-being |
| **Health literacy** | Health literacy (parents, children, teachers…), knowledge and use of available services |
| **Health promotion in (pre)schools** | Health promotion curriculum, participation to health promotion programs |
| **Utilization of health care/educational services** | Screening examination utilization, immunization status |
| **Public health service^1^ indicators** | (Pre)school entry health checks data, child development, child dental health |
| **Nutritional status and behavior** | Abdominal girth, soft drinks consumption |
| **Sexual health** | Contraception use, access to contraceptives |
| **Sleep health** | Sleeping disorders, sleeping behavior |
| **Addictions** | Addictive substances use |
| **Media use** | Media use duration |
| **Violence related indicators** | Child neglect, child dental neglect, domestic violence |
| **Inclusion** | Availability of inclusive services, employment rate of people with disabilities |
| **Other health status indicators** | Anxiety disorders, suicides, injuries |
| **Index combining diverse topics** | “Health challenges index” ^2^ |

*Notes.* ^1^ Public health service: “öffentlicher Gesundheitsdienst, ÖGD“ in German. ^2^ Index "Gesundheitliche Herausforderung" in German.
